# The administration of antisense oligonucleotide golodirsen reduces pathological regeneration in patients with Duchenne muscular dystrophy

**DOI:** 10.1186/s40478-020-01106-1

**Published:** 2021-01-06

**Authors:** Dominic Scaglioni, Francesco Catapano, Matthew Ellis, Silvia Torelli, Darren Chambers, Lucy Feng, Matthew Beck, Caroline Sewry, Mauro Monforte, Shawn Harriman, Erica Koenig, Jyoti Malhotra, Linda Popplewell, Michela Guglieri, Volker Straub, Eugenio Mercuri, Laurent Servais, Rahul Phadke, Jennifer Morgan, Francesco Muntoni

**Affiliations:** 1 Dubowitz Neuromuscular Centre, UCL Great Ormond Street Institute of Child Health, 30 Guilford St, London, WC1N 1EH UK; 2 NIHR Great Ormond Street Hospital Biomedical Research Centre, UCL Great Ormond Street Institute of Child Health and Great Ormond Street Hospital for Children NHS Foundation Trust, London, UK; 3 Dubowitz Neuromuscular Centre, UCL Queen Square Institute of Neurology and Great Ormond Street, London, UK; 4 Department of Neurodegenerative Diseases, UCL Queen Square Institute of Neurology, London, UK; 5 School of Cancer Sciences, University of Southampton, Southampton, UK; 6 RJAH Orthopaedic Hospital NHS Trust, Oswestry, UK; 7 Paediatric Neurology and Centro Clinico Nemo, Catholic University and Policlinico Gemelli, Fondazione Policlinico Universitario Agostino Gemelli IRCSS, Rome, Italy; 8 Sarepta Therapeutics, Inc., Cambridge, MA USA; 9 Centre of Gene and Cell Therapy and Centre for Biomedical Sciences, Royal Holloway, University of London, Egham, UK; 10 Newcastle University John Walton Muscular Dystrophy Research Centre and the Newcastle Hospitals NHS Foundation Trust, Newcastle upon Tyne, UK; 11 Institute I-Motion, Hôpital Armand-Trousseau, Paris, France; 12 Neuromuscular Reference Centre, CHU Liège, Liège, Belgium

**Keywords:** Dystrophin, Muscular dystrophy, Immunofluorescence, Genetic therapies, Golodirsen, Clinical trial

## Abstract

During the last decade, multiple clinical trials for Duchenne muscular dystrophy (DMD) have focused on the induction of dystrophin expression using different strategies. Many of these trials have reported a clear increase in dystrophin protein following treatment. However, the low levels of the induced dystrophin protein have raised questions on its functionality. In our present study, using an unbiased, high-throughput digital image analysis platform, we assessed markers of regeneration and levels of dystrophin associated protein via immunofluorescent analysis of whole muscle sections in 25 DMD boys who received 48-weeks treatment with exon 53 skipping morpholino antisense oligonucleotide (PMO) golodirsen. We demonstrate that the de novo dystrophin induced by exon skipping with PMO golodirsen is capable of conferring a histological benefit in treated patients with an increase in dystrophin associated proteins at the dystrophin positive regions of the sarcolemma in post-treatment biopsies. Although 48 weeks treatment with golodirsen did not result in a significant change in the levels of fetal/developmental myosins for the entire cohort, there was a significant negative correlation between the amount of dystrophin and levels of regeneration observed in different biopsy samples. Our results provide, for the first time, evidence of functionality of induced dystrophin following successful therapeutic intervention in the human.

**Electronic supplementary material:**

The online version of this article (10.1186/s40478-020-01106-1) contains supplementary material, which is available to authorized users.

## Background

Duchenne muscular dystrophy (DMD) is a genetic, X-linked, muscle-wasting disorder that affects 1:3500–5000 boys and is caused by mutations in the *DMD* gene [24]. The mutations found in DMD patients disrupt the open reading frame of the gene resulting in the inability to produce the protein dystrophin [30]. The lack of dystrophin triggers progressive muscle degeneration, leading to loss of muscle tissue and progressive weakness, with loss of ambulation by early teens, and ultimately premature death [48]. The primary aim of many DMD clinical trials is the induction of dystrophin and several DMD therapies have now successfully induced the production of low levels of dystrophin protein with correct localisation at the sarcolemma [7, 11, 19, 46]. However, the question surrounding the molecular functionality of de novo dystrophin protein in humans as a result of therapeutic intervention, and, in particular, whether the low levels of protein expression can provide functional benefit to the DMD muscle has not been assessed before.

Dystrophin is an essential protein localised to the cytoplasmic face of the sarcolemma that connects intracellular cytoskeletal actin of muscle cells to the extracellular matrix (ECM) and these interactions are critical for sarcolemmal membrane stability. This cross sarcolemmal linkage occurs via dystrophin’s interaction with a myriad of binding partners both at the sarcolemma and within the cytoplasm [23]. At the sarcolemma, dystrophin links a complex of proteins that together assist the connection of the ECM to the cytoskeleton, resulting in membrane stabilisation during muscle contractions [10, 15]. This dystrophin-associated protein complex (DAPC) includes the dystroglycans (α/β), sarcoglycans (α, β, γ, δ), syntrophins, dystrobrevins and others [16]. Pathological consequences caused by the lack of functional dystrophin and the subsequent reduction in the DAPC are dramatic, with a progressive cascade of events, including repeated cycles of myofibre degeneration and regeneration, resulting in the re-expression of immature myosin heavy chain isoforms in regenerating myofibres [14, 21]. This muscle degeneration starts at birth and in the initial phases of the condition is compensated by an effective regeneration which eventually fails to compensate for the degeneration, leading to replacement of myofibres with fibrofatty and connective tissue over time. Preventing or reducing these cycles of myofibre degeneration/regeneration can reduce pathological inflammation, fibrosis, and preserve muscle bulk and function [32, 41]

Mutations in *DMD* that maintain the reading frame result in a milder phenotypic form of the disease, Becker muscular dystrophy (BMD) [26]. BMD patients typically have more dystrophin and DAPC proteins with fewer regenerating myofibres and this results in less progressive pathology and improved muscular and ambulatory capacity [18, 25, 40]. BMD therefore represents the proof of concept that reframing the dystrophin transcript using antisense oligonucleotides to produce internally deleted dystrophin protein should alleviate the disease severity observed in patients with DMD [2, 35, 39, 43].

Golodirsen (formerly SRP-4053 and now commercially Vyondys 53™) is a phosphorodiamidate morpholino oligomer (PMO) that specifically targets exon 53 of dystrophin pre-mRNA, resulting in its exclusion from the final mRNA product [19]. Skipping of exon 53 in DMD patients with amenable mutations results in restoration of the mRNA reading frame and leads to the production of a partially internally deleted dystrophin protein with intact C and N-terminal regions [19]. Roughly 7.7% of DMD patients have mutations that can have their reading frame restored by skipping of exon 53 [1]. We previously showed that following 48-weeks treatment with golodirsen, there was a statistically significant increase in dystrophin protein compared to baseline measurements in 25 DMD boys. Increased dystrophin expression was determined via validated, non-normalized western blot, which showed a mean percent to normal control pool dystrophin protein standard of 1.019% (range 0.09–4.30%) and an increase in mean fluorescent intensity. Correct sarcolemmal localisation was also shown via immunofluorescent quantification of the percentage of dystrophin positive myofibres [19]. However, it is also important to evaluate the molecular functionality of such dystrophin by demonstrating that this induced protein correctly interacts with the DAPC– a feature crucial to preserving the sarcolemmal stability during myofibre contraction.

In the present analysis, we quantified the colocalised expression of dystrophin and key DAPC proteins in clinical trial samples following 48 weeks of therapeutic intervention with golodirsen. Furthermore, using our recently published unbiased high-throughput digital script [40], we determined for the first time if the levels of dystrophin produced was sufficient to reduce the amount of degeneration in the muscle of the treated patients, an essential assessment of the molecular functionality of the induced dystrophin [9, 31, 34].

## Methods

### Patient demographics

All work in this study was performed as further analysis of the 4053-101 study (NCT02310906). Boys with a confirmed DMD diagnosis aged 6–15 years with mutations amenable to correction with exon 53 skipping were recruited. Full patient demographics and mutation status are listed in Table 1.

**Table 1:** Patient demographics and mutations

Characteristics	All patients (N = 25)
Age (years)	8.2 (2.2)
Height (cm)	120.1 (10.4)
Weight (kg)	28.2 (9.1)
BMI (kg/m^2^)	19.1 (3.7)
6MWT distance (m)	403.7 (56.7)
Time since DMD diagnosis (MO)	55.2 (24.9)
Duration of corticosteroid use (MO)	36.8 (25.9)

Patient Id	Mutation
1	∆49–52
2	∆45–52
3	∆45–52
4	∆45–52
5	∆49–52
6	∆48–52
7	∆48–52
8	∆45–52
9	∆48–52
10	∆50–52
11	∆50–52
12	∆49–52
13	∆45–52
14	∆45–52
15	∆49–52
16	∆52
17	∆50–52
18	∆45–52
19	∆45–52
20	∆48–52
21	∆49–52
22	∆50–52
23	∆48–52
24	∆52
25	∆52

6MWT 6-minute walk test, BMI body mass index, DMD Duchenne muscular dystrophy

Values are shown as mean (SD)

∆ deletion of exons

### Muscle sectioning

Muscle biopsies were collected from one biceps brachii muscle at baseline and then from the contralateral biceps following part 2 of the NCT02310906 study after 48 weeks. Serial unfixed frozen Sects. (5 μm-thick) were cut from baseline and 48 weeks muscle biopsies from all 25 boys involved in the SKIP-NMD 4053-101 trial using a Leica CM 1850 UV cryostat (Leica Biosystems, Germany). 3 × serial sections were collected on each slide and slides were stored at − 80 °C until time of use. Only 1 muscle sample from each biopsy was analysed for this study as previous work found no difference in dystrophin expression between two adjacent muscle biopsy (samples A and B) from the original reporting [19].

### Sample blinding

All biopsy samples were blinded for patient ID and biopsy time point at the time of cryosectioning. Tissue sections were assigned blinding codes generated and provided by PharPoint Research, Inc. (Durham, NC). Cryosectioning and blinding were performed by an independent operator who was not involved in further experiments or subsequent data analysis. Individuals performing the experiments were blinded until all data had been acquired. Unblinding was then performed to allow for statistical analysis and comparison of baseline and 48-week results.

### Immunostaining

Slides were removed from − 80 °C storage and air-dried for 45 min before staining. A Super pap pen (Daido Sangyo LTD, Japan) was used to create hydrophobic barriers around the tissue. Samples were incubated with 200–300 μl of primary (1 h-RT) and 200–300 μl of secondary (30 min-RT) antibodies diluted in PBS (Fisher Scientific, UK). Volumes varied based on the size of the tissue sections to ensure they were completely covered. Details of the antibody combinations and specifications used are listed in Table 2. A cocktail of developmental and fetal/developmental myosin antibodies (designated f/d myosin cocktail) was employed to detect the majority of fibres in different stages of regeneration or aberrant re-expression of these immature myosin heavy chain isoforms in dystrophic myofibres. Sections were washed for 3 × 3 min with PBS, before and after the incubation with secondary antibodies. Stained sections were mounted with Hydromount (National Diagnostics, UK) and coverslipped with cover glasses (VWR, Belgium). Slides were protected from light and stored at 4 °C until acquisition. For each sample, 3 serial sections on the same slide were stained in triplicate for each experiment.

**Table 2:** All antibodies used with information on species, isotype, class, catalogue number and working dilution. Figure shows antibody combinations used for (**a**) α-sarcoglycan (**b**) β-dystroglycan and (**c**) f/d myosin triple stains

Primary antibody	Species	Class	Catalogue #	Dilutions
β-dystroglycan	Mouse IgG2a	Monoclonal	NCL-b-DG	1:20
Dystrophin (exon 77)	Rabbit IgG	Polyclonal	ab15277	1:200
Laminin α2-1 (300 kDa)	Rat IgG1	Monoclonal	804-190-C100	1:50
Myosin developmental	Mouse IgG1	Monoclonal	NCL-MHCd	1:30
Myosin neonatal	Mouse IgG1	Monoclonal	NCL-MHCn	1:30
α-sarcoglycan	Mouse IgG1	Monoclonal	NCL-a-SARC	1:40
Secondary antibody	Species	Class	Catalogue #	Dilutions
Alexa Fluor 488	Goat anti-Rabbit IgG	Polyclonal	A-11034	1:100
Alexa Fluor 568	Goat anti-Rat IgG	Polyclonal	A-11077	1:100
Alexa Fluor 647	Donkey anti-Mouse IgG	Polyclonal	A-31571	1:100

### Acquisition

All slides were scanned within 24 h of immunostaining on a ZEISS Axio Scan.Z1 Slide Scanner (Carl Zeiss Microscopy GmbH, Germany). Laminin α2 staining in the 568 channel was used as a reference marker protein for coarse and fine focus map generation using 10 × and 20 × objectives, respectively. Following focus map creation, 3 fluorescent channel (488, 568 and 647) whole slide images were captured using the 20 × objective with offline image stitching for greater accuracy. The slide scanner is calibrated annually with its LEDs measured to an internal reference standard to ensure stable and constant output power over the entire lifetime of each LED.

### Digital image analysis

The analysis was implemented using Definiens Developer XD (Munich), version 2.7.0. A detailed review of the image analysis platform has previously been published [40].

In brief, the image processing is comprised of three distinct stages: identification of muscle tissue boundary; identification of transverse myofibres within the tissue (longitudinal myofibres are excluded); definition of sarcolemmal and sarcoplasmic regions; and finally, characterisation of morphological features and immunostaining profiles of individual muscle fibres for specific markers.

For sarcolemmal staining, a dynamic background subtraction method was used to identify positive staining above the Gaussian smoothed stain topography. Myofibres were classed as sarcolemmal protein positive if they contained greater than 25% sarcolemmal circumference coverage of positive protein staining. For f/d myosin classification, myofibres were designated positive if they had average sarcoplasm fluorescence intensity greater than a negative threshold value that was determined from f/d myosin staining of CTRL samples (where positive myofibres are not routinely expected).

### Statistical analysis

Data for each section were generated from individual analysis of every successfully identified myofibre within that section. This ranged from a few hundred myofibres to many thousands, depending on the size of the section. Where possible, data from 3 sections for each sample was used for final analysis. If a section was compromised due to artefacts in the original immunostained image or during the automated image analysis, it was excluded and data from 2 sections used. If 2 sections were compromised, data from the final remaining section alone was used, as this was still generated from analysis of many individual myofibres within the section. If all 3 sections from a given sample were significantly impaired, that sample was excluded from the analysis.

Comparisons of baseline vs 48 week time points was computed using a paired *T* test. Correlations were performed assuming a non-Gaussian distribution and computing Spearman correlation coefficient. Statistical significance refers to *p* < 0.05

Graphs are presented as mean $\pm$ standard deviation unless otherwise stated.

## Results

3 × serial sections of baseline and 48-week biopsy samples from all 25 patients previously acquired in the SKIP-NMD 4053-101 study (NCT02310906) were immunostained for laminin α2, dystrophin and a tertiary protein marker (α-sarcoglycan/β-dystroglycan or f/d myosin).

The levels of dystrophin and these tertiary protein markers were then quantified at both time points via digital image analysis. A variety of parameters were then assessed for the tertiary protein markers including changes in sarcolemmal fluorescence intensity, sarcolemmal fluorescence intensity in dystrophin positive and dystrophin negative sarcolemmal regions,  % positive myofibres for each protein and finally their correlation with levels of dystrophin.

### Assessment of dystrophin-associated proteins (DAPs)

**Fig. 1:**
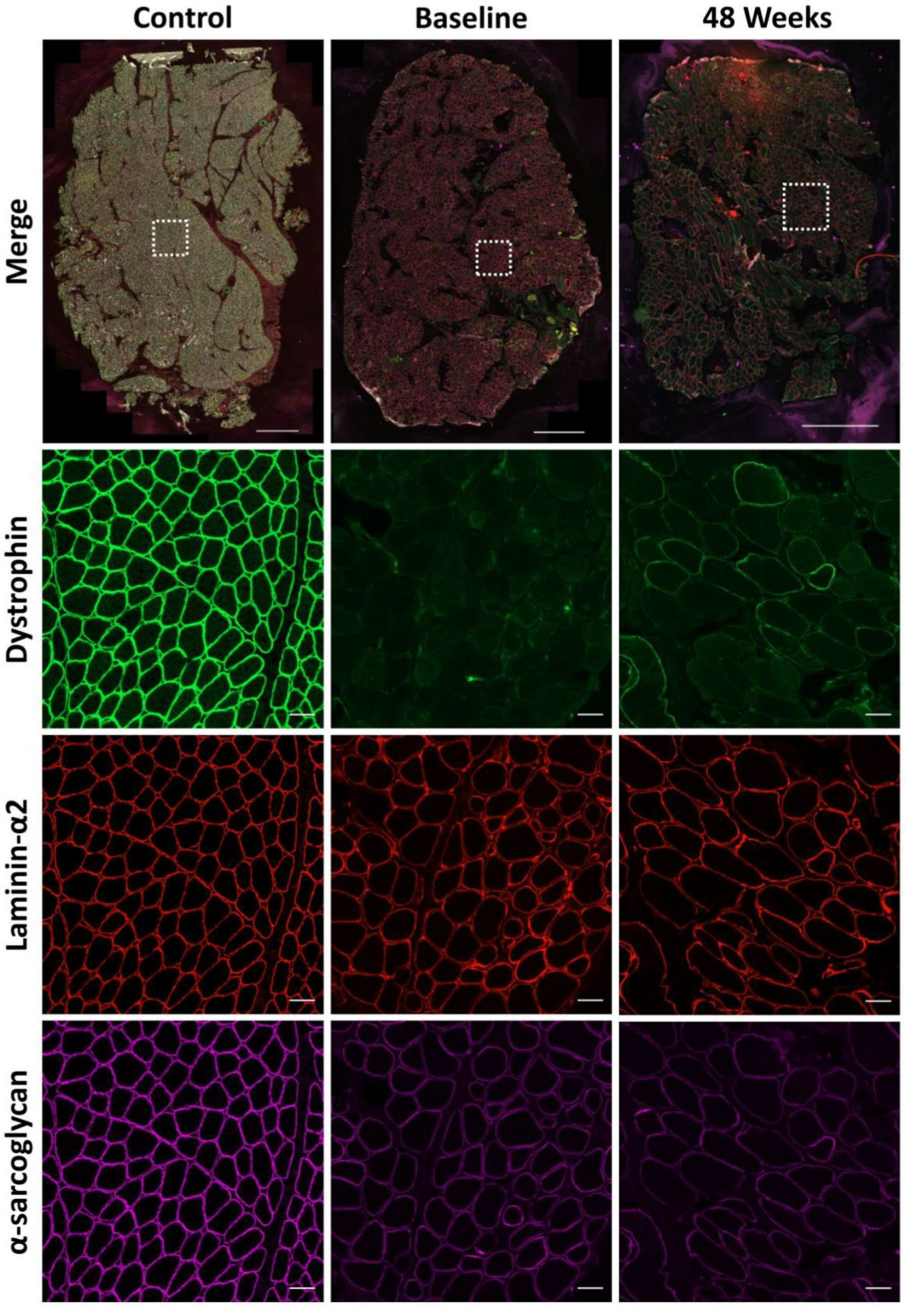
Representative example of a control section along with sections at baseline and 48 weeks from 1 patient immunostained for dystrophin (green, 488), laminin-α2 (red, 568) and α-sarcoglycan (purple, 647). Scale bar of merged whole section image = 1000 µm. Scale bar for the regions of interest is 50 µm

**Fig. 2:**
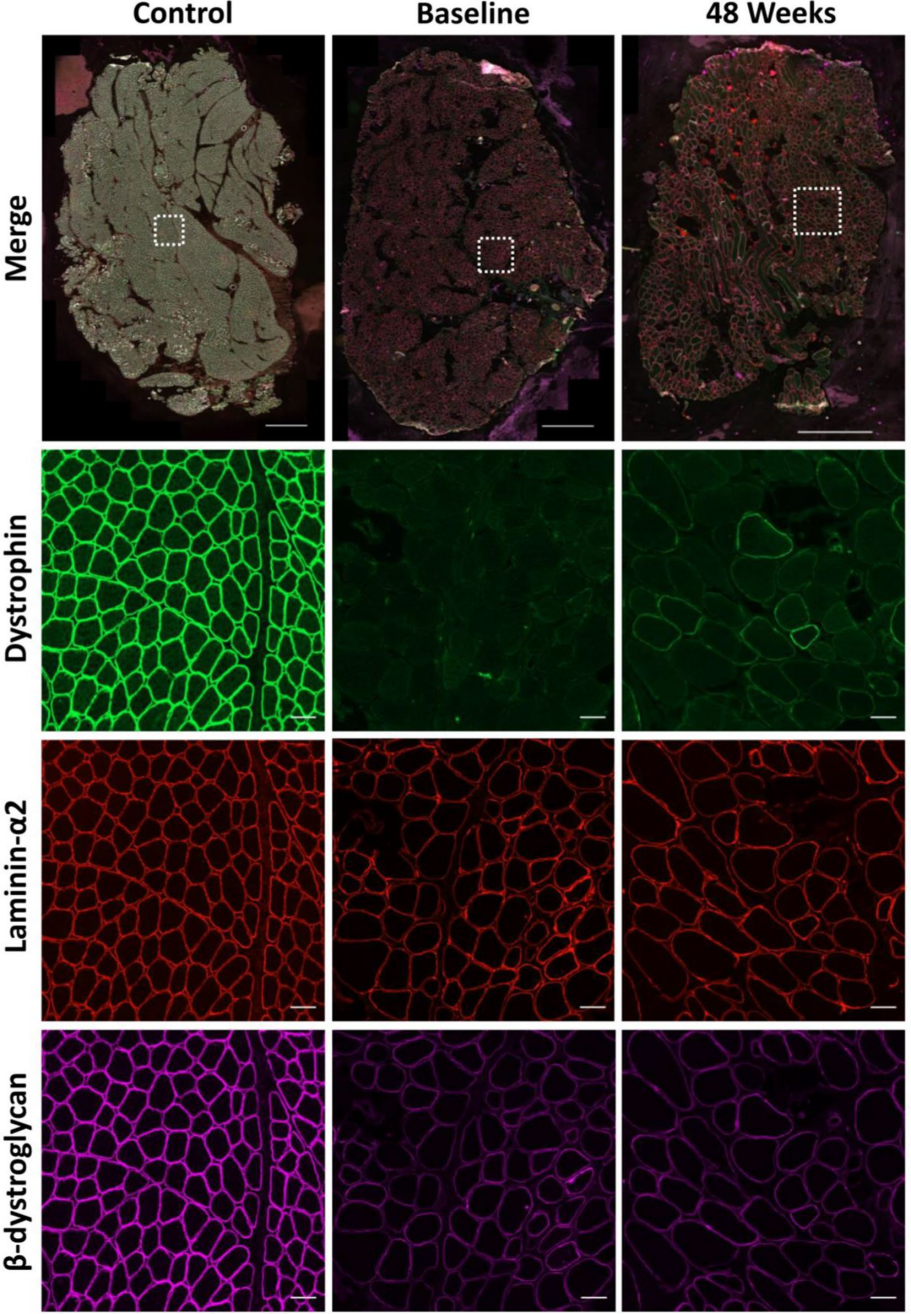
Representative example of a control section along with sections at baseline and 48 weeks from 1 patient immunostained for dystrophin (green, 488), laminin-α2 (red, 568) and β-dystroglycan (purple, 647). Scale bar of merged whole section image = 1000 µm. Scale bar for the regions of interest is 50 µm

We analysed the absolute and dystrophin colocalised quantification of two key DAPC proteins following treatment with golodirsen. Baseline and post-treatment biopsies were immunostained for laminin-α2, dystrophin and either α-sarcoglycan or β-dystroglycan. A representative example of a control section along with sections from a baseline and 48 week sample immunostained for α-sarcoglycan and β-dystroglycan can been seen in Figs. 1 and 2, respectively. Both of these proteins are essential for the stabilisation of the sarcolemma and prevention of contraction-induced muscle damage. We then looked at the changes in sarcolemmal fluorescence intensity of the DAPs between the two treatment time points and investigated their relationship with levels of dystrophin. Baseline samples from patients 22 and 24 and the 48-week sample from patient 21 were excluded as the sections were unsuitable for accurate automated analysis (images available for inspection on request).

#### Changes in sarcolemmal DAP fluorescent intensity

**Fig. 3:**
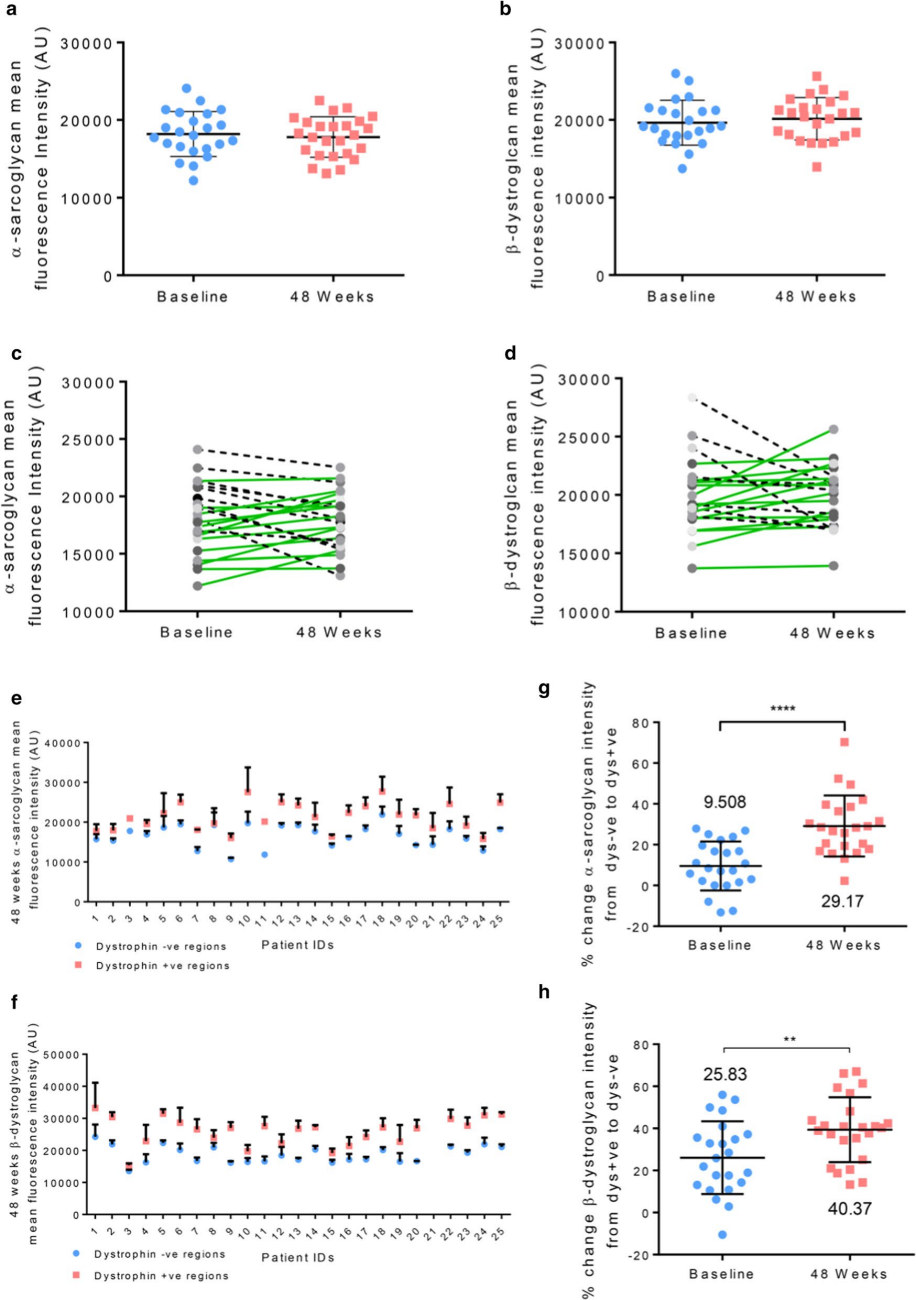
Mean fluorescent intensity of total sarcolemmal α-sarcoglycan (**a**) and β-dystroglycan (**b**) at baseline and following 48 weeks therapeutic intervention with golodirsen. Each data point represents the average intensity for each patient at each time point. Changes in α-sarcoglycan (**c**) and β-dystroglycan (**d**) sarcolemmal fluorescent intensity for each patient from baseline to 48 weeks. α-Sarcoglycan (**e**) and β-dystroglycan (**f**) fluorescent intensity after 48 weeks treatment in regions of dystrophin positive and dystrophin negative sarcolemma. Percentage change in α-sarcoglycan (**g**) and β-dystroglycan (**h**) intensity from dystrophin negative to dystrophin positive sarcolemma at both baseline and 48 week time points

When analysing the whole section average of all patients, we found no significant change in levels of either β-dystroglycan or α-sarcoglycan following treatment. Fluorescence intensity values for α-sarcoglycan were 18188AU (arbitrary units) and 17794AU (Fig. 3a) at baseline and 48-weeks respectively while the values for β-dystroglycan were 19646AU and 20143AU (Fig. 3b). We also saw no significant change in the  % of myofibres positive for either DAP between the two time points, due to the fact that most fibres were already positive in the baseline muscle biopsy. Indeed, baseline and 48-week values for α-sarcoglycan positive myofibres were 97% and 98% respectively whilst values for β-dystroglycan were 98% at both time points.  % positive myofibres for α-sarcoglycan and β-dystroglycan at baseline and 48-weeks for all patients can be seen in Additional file 1: Fig. S1.

12 patients (55%) showed an increase in α-sarcoglycan intensity (Fig. 3c) following treatment whilst 10 (45%) saw a decrease. For β-dystroglycan (Fig. 3d), we demonstrated that 14/22 (64%) patients had an increase in global intensity from baseline to 48 weeks. In 8/22 (36%) we detected a decrease in intensity between the 2 time-points. Results from all patients, including numerical change in intensity and  % change between the 2 time points for both proteins, can be seen in Additional file 1: Fig. S2.

#### DAP fluorescence intensity in dystrophin +ve/−ve sarcolemmal regions

We then assessed the fluorescence intensity of α-sarcoglycan and β-dystroglycan in the post-treatment biopsies by comparing the intensity of these DAPs in discrete regions of the sarcolemma that were classified as either dystrophin positive or dystrophin negative. The classification was performed utilising the same method published in our previous manuscript [40]. In all samples, regions of the sarcolemma that were dystrophin positive had greater α-sarcoglycan (Fig. 3e) and β-dystroglycan (Fig. 3f) intensity compared to sarcolemmal regions where dystrophin was not present.

We then compared the  % change in α-sarcoglycan and β-dystroglycan intensity from dystrophin negative to dystrophin positive sarcolemmal regions in both baseline and 48-week biopsies. Following treatment, the  % change in α-sarcoglycan (Fig. 3g) and β-dystroglycan (Fig. 3h) intensity between dystrophin positive and dystrophin negative sarcolemma was significantly greater than the difference at baseline.

#### Correlation between levels of dystrophin and DAP

**Fig. 4:**
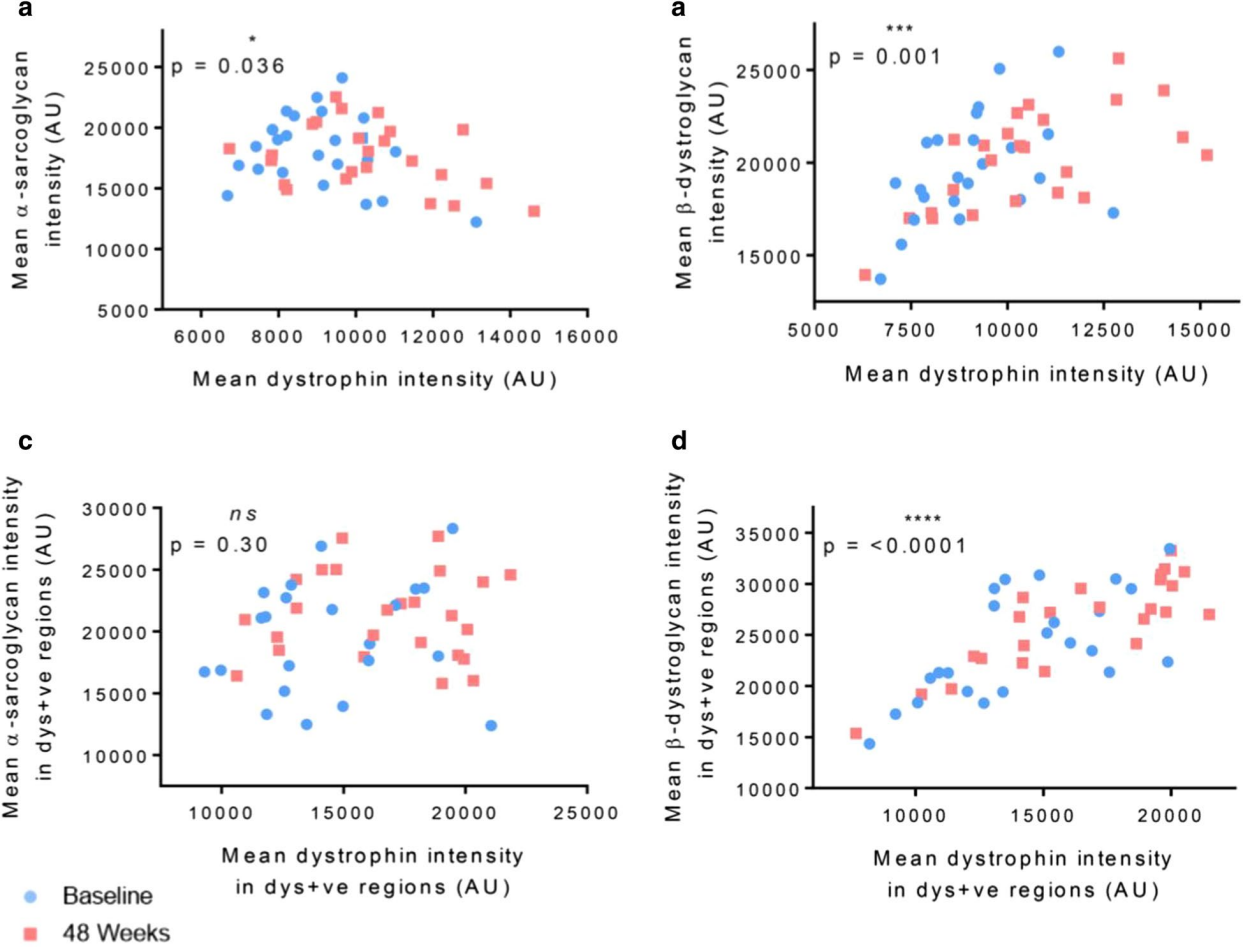
Correlation between mean sarcolemmal fluorescence intensity in the dystrophin channel and mean sarcolemmal α-sarcoglycan (**a**) and β-dystroglycan (**b**) intensity. Relationship between mean dystrophin intensity in regions of dystrophin positive sarcolemmal and α-sarcoglycan (**c**) or β-dystroglycan (**d**) fluorescence intensity in those corresponding regions of dystrophin positivity

Finally, we assessed the correlation between the amount of α-sarcoglycan/β-dystroglycan and the amount of dystrophin in both baseline and 48-week biopsies. First, we compared simply the average sarcolemmal fluorescence intensity for α-sarcoglycan (Fig. 4a) and β-dystroglycan (Fig. 4b) against the average sarcolemmal intensity of dystrophin. For α-sarcoglycan, a non-significant correlation existed (*p* = 0.12, r = − 0.23) where seemingly higher levels of dystrophin were not associated with an increase in α-sarcoglycan intensity. In comparison, the correlation of β-dystroglycan intensity against dystrophin revealed a stronger and significant positive correlation (*p* < 0.0001, r = 0.55). An increase in dystrophin mean sarcolemmal intensity was associated with an increase in mean β-dystroglycan intensity.

Following this, we looked more closely at the relationship between dystrophin positive regions of the sarcolemma and the fluorescence intensity of the DAPs in those specific sarcolemmal regions. When categorising intensity based on discrete regions of the sarcolemma that were dystrophin positive, we saw a positive correlation for both α-sarcoglycan (Fig. 4c) and β-dystroglycan (Fig. 4d). Despite this stratification, the correlation for α-sarcoglycan was still not significant. In comparison, for β-dystroglycan, a strongly significant positive correlation (*p* < 0.0001, r = 0.7) was again confirmed. While there is heterogeneity between the distribution of baseline and 48-week samples, there is a clear grouping of post-treatment samples with high levels of both mean dystrophin intensity in dystrophin positive regions and mean β-dystroglycan intensity in dystrophin positive sarcolemmal areas. In comparison, the majority of samples with low mean dystrophin and β-dystroglycan intensity were from baseline biopsies.

### Myofibre regeneration

#### % f/d myosin positive fibres

**Fig. 5:**
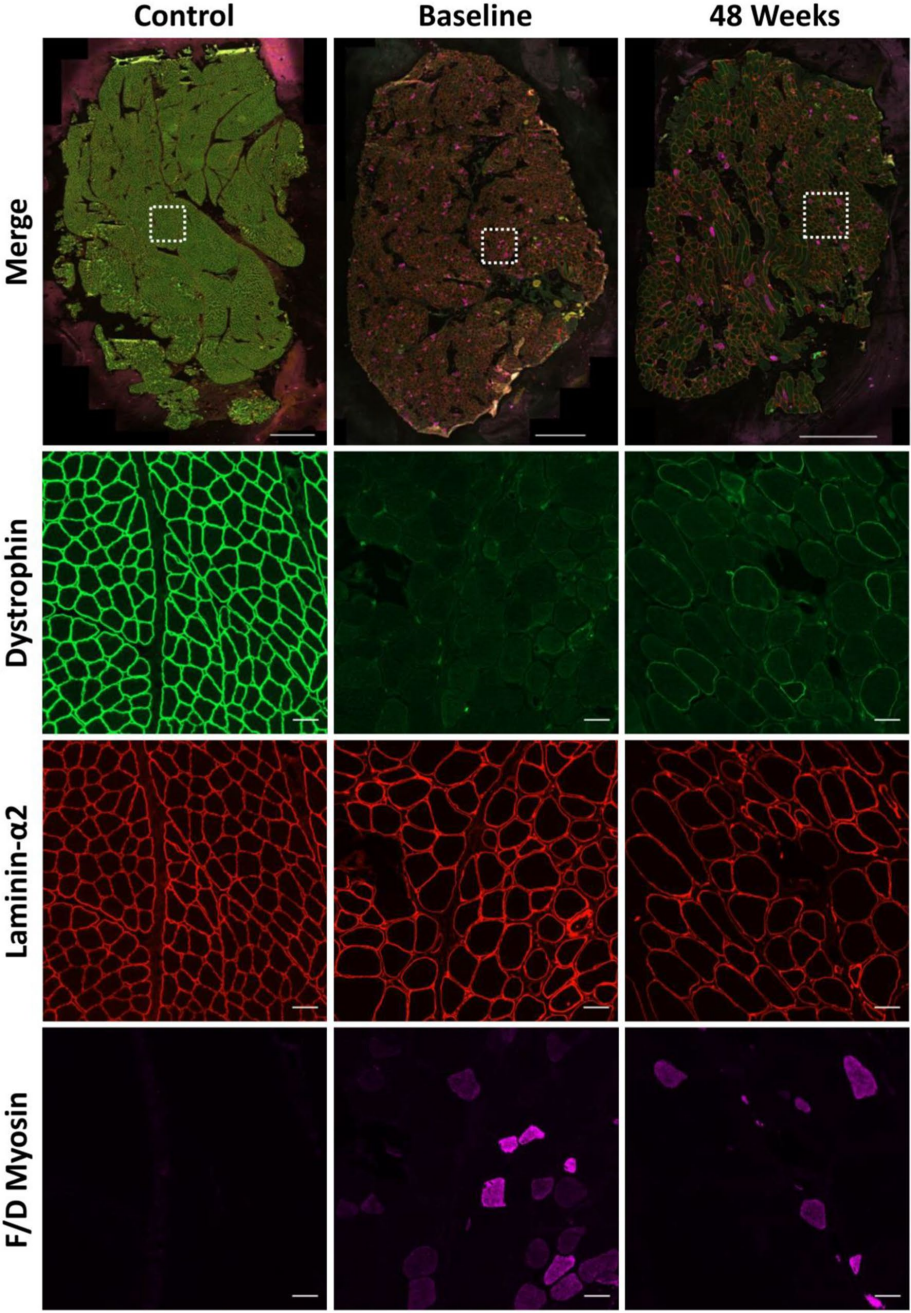
Representative example of a control section along with sections at baseline and 48 weeks from 1 patient immunostained for dystrophin (green, 488), laminin-α2 (red, 568) and f/d myosins (purple, 647). Scale bar of merged whole section image = 1000 µm. Scale bar for the regions of interest is 50 µm

A cocktail of antibodies against fetal and developmental immature myosin isoforms (f/d myosin) was used to identify myofibres in various stages of regeneration and fibres with an induced expression as a marker of progressive pathology. A representative example of a control section along with sections from a baseline and 48 week sample immunostained for dystrophin, laminin-α2 and F/D myosins can been seen in Fig. 5. Within each section, the number of f/d myosin positive fibres was determined, and a percentage positivity value generated by the automated script based on the total number of myofibres identified in each section.  % positivity was used to allow for correlation of biopsies that varied considerably in size and the absolute number of myofibres identified. Results from one patient (patient 22) were excluded from analyses due to having a baseline biopsy that was very small and unsuitable for automated digital assessment (images of the baseline biopsy available on request).

**Fig. 6:**
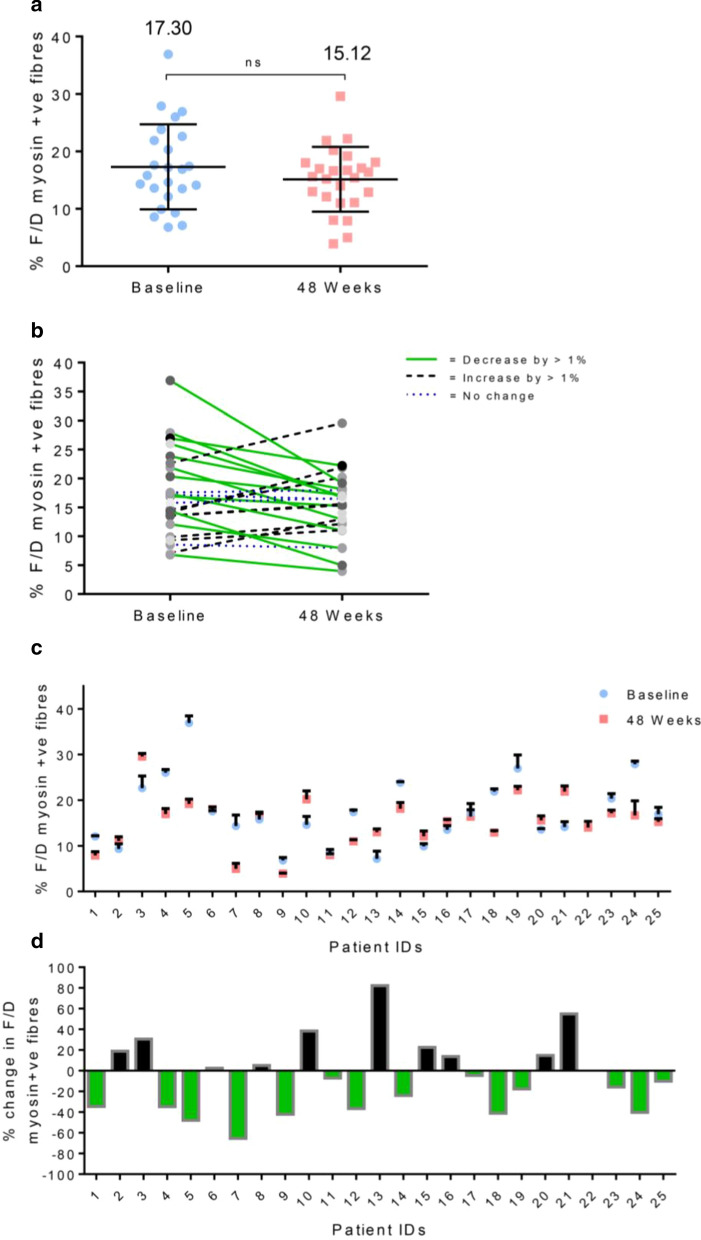
(**a**) Mean percentage of fetal and developmental (f/d) myosin positive fibres at baseline and 48-week time points for all patients. (**b**) Percentage of f/d myosin positive fibres at baseline and 48 weeks for each patient. (**c**) Percentage f/d positive myofibres at baseline and 48 weeks for all 25 patients. Baseline sample from patient 22 was excluded due to the biopsy being unsuitable for automated digital analysis. (**d**)  % change in f/d myosin positive fibres from baseline to 48 weeks for each patient

The mean percentage f/d myosin positive fibres of all patients were 17.3% (14.2, 20.4 CI) at baseline compared to 15.1% (12.8, 17.5 CI) at 48 weeks. While this difference was not statistically significant, it does represent a 2.2% decrease between the two time points. (− 5.9, 1.6 CI). (Figure 6a).

The changes in  % of f/d myosin positive fibres were highly variable across the entire 25 patient cohort. In 12/24 patients (50%) there was a decrease (> 1%) in  % f/d myosin positive fibres between the two time points (Fig. 6b). The average decrease for these 12 patients was 7.1% (Additional file 1: Fig. S3a). 8/24 patients (33%) saw an increase in regenerating myofibres from baseline to 48 weeks while in 4/24 (17%) there was minimal difference (less than 1% in either direction) between time points (Fig. 6b). The average increase for these 8 patients was 4.3% (Additional file 1: Fig. S3b).

Results from all patients can be seen in Fig. 6c. The greatest decrease was observed in patient 5 who had a 17.7% reduction in the percentage of f/d myosin positive fibres in the biopsy. This equates to a 1.9-fold reduction from baseline. Patient 7 exhibited the greatest fold change reduction (2.9-fold) with baseline and 48-week values of 14.3% and 5% respectively (Fig. 6d). The greatest increase was from patient 21 whose  % f/d myosin fibres went from 14.1 to 21.9% following treatment, representing a 1.5-fold increase. Patient 13 saw the greatest fold-change with baseline and 48-week values of 7.1% and 13.0%, representing a 1.8-fold increase in f/d myosin positive fibres between the two time points.

#### Correlation between dystrophin and levels of regeneration

We next assessed the correlation between levels of dystrophin (measured as both mean sarcolemmal fluorescence intensity and  % dystrophin positive fibres) and  % of f/d myosin positive fibres in baseline and 48-week biopsy samples.

**Fig. 7:**
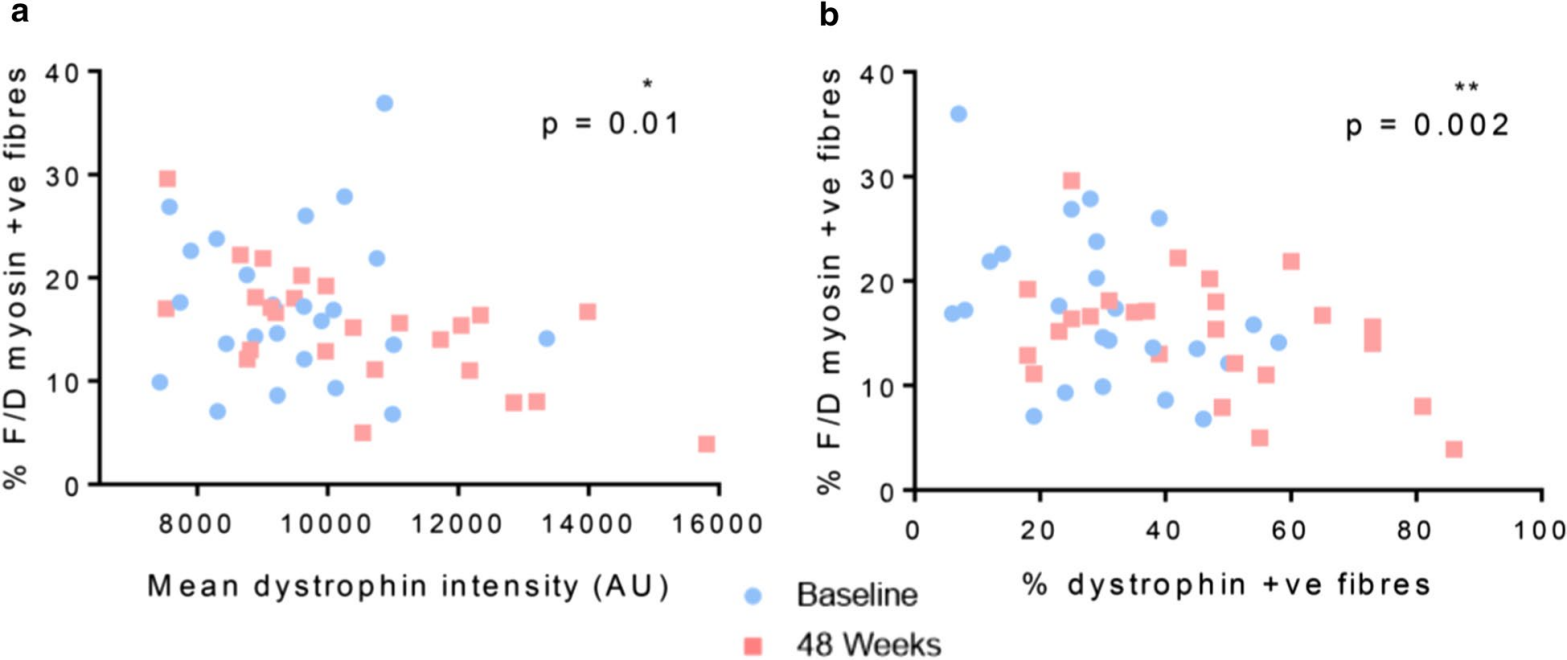
Correlation between mean dystrophin intensity (**a**) and  % dystrophin positive myofibres (**b**) against percentage of f/d myosin positive fibres

There was a significant negative correlation between  % f/d myosin positive fibres and both sarcolemmal dystrophin intensity (r = −  0.4, *p* < 0.05) (Fig. 7a) and  % dystrophin positive fibres (r = −  0.4, *p* < 0.005) (Fig. 7b). Lower levels of  % f/d myosin positive fibres were associated with higher levels of dystrophin when quantified by both methods. While there was heterogeneity in the distribution of baseline and 48-week samples, there is a clear grouping of post treatment samples (Fig. 7a, b) with greater levels of dystrophin and lower levels of f/d myosin positive fibres compared to the majority of baseline samples, which predominantly present with lower levels of dystrophin and elevated f/d myosin positive myofibres.

Additionally, there was a significant correlation between  % myosin +ve fibres at 48 weeks and the  % change in dystrophin intensity from baseline to 48 weeks (r = −  0.6, *p* < 0.005; Additional file 1: Fig. S3b). However, this correlation was not maintained when plotting  % change of dystrophin positive fibres against  % f/d myosin positive fibres (Additional file 1: Fig. S3c).

## Discussion

Several recent clinical trials have demonstrated the possibility of inducing the production of low levels of dystrophin in boys with DMD following either small molecule therapy [17] or AONs targeted to induce skipping of exon 51 (eteplirsen; drisapersen) or skipping of exon 53 (vitolarsen; golodirsen). While in a few of these studies the restoration of DAPC proteins was demonstrated, these studies did not systematically assess the impact of this restoration on additional aspects of muscle pathology, including myofibre regeneration [7–9, 11, 19, 29, 46].

The ability of the low levels of induced dystrophin to reduce the susceptibility to myofibre necrosis and the subsequent cycles of degeneration and regeneration has been previously demonstrated in the *mdx* mouse preclinical model following AON therapies [22], and following AAV gene therapy [13, 45, 49]. However, whether the low levels of dystrophin observed in the human trial can also exert a positive effect on the progression of the muscle pathology is currently unknown. As there is a clear relationship between the levels of dystrophin expression and the levels of regeneration in patients with BMD [25, 40] and in view of the findings of decrease degeneration/regeneration in DMD preclinical models [37, 47], we decided to investigate the surrogate molecular functionality of induced dystrophin in humans following a 48-week period of therapeutic intervention with exon 53 skipping PMO golodirsen. Using whole section images of fluorescently stained muscle biopsy sections and automated, unbiased image analysis software, we sought to determine if induced dystrophin following 48 weeks treatment was sufficient to enhance levels of dystrophin-associated proteins in regions of dystrophin positive sarcolemma and alter the extent of myofibre regeneration.

### Localised increase in dystrophin associated proteins

A novel aspect of our methodology is the ability to assess colocalisation data of 2 sarcolemmal proteins, thus enabling the correlation of relevant DAPC proteins with discrete sarcolemmal regions that are either dystrophin positive or dystrophin negative. AON treatment results in a ‘patchy’ distribution of dystrophin with varying expression patterns not only between myofibres but also at the sarcolemma of individual myofibres. As such, when assessing molecular efficacy of the protein, it is vital to distinguish between these divergent dystrophin positive and negative sarcolemmal regions. When specifically assessing dystrophin positive portions of the sarcolemma, we demonstrated a greater amount of both α-sarcoglycan and β-dystroglycan in all patients compared to dystrophin-negative sarcolemma, indicating that the induced dystrophin recruits the DAPC more efficiently.

Despite this increase in dystrophin positive sarcolemmal regions, when assessing the global sarcolemmal change for both α-sarcoglycan and β-dystroglycan in the entire cohort of patients, there was no significant alteration in fluorescence intensity between the two time points. We interpreted these findings as a result of the heterogeneous response to the drug, with some patient having a relatively high number of dystrophin positive fibres, while others only demonstrating a small increase after 48-weeks treatment, which failed to sufficiently alter baseline DAPC levels [19]

When assessing the correlation between the amount of dystrophin and DAPs, the correlation of dystrophin with β-dystroglycan was seemingly stronger than it was for α-sarcoglycan. This result is reflective of previous work where a significant positive correlation between utrophin and β-dystroglycan was observed but this correlation was not maintained between utrophin and another protein of the complex, γ-sarcoglycan [25]. Dystrophin is known to directly interact with β-dystroglycan, while the sarcoglycans do not directly bind dystrophin [12, 20]. It is plausible that this interaction is causative of the differing correlations that we observed between dystrophin with α-sarcoglycan and β-dystroglycan, and indeed we previously observed in BMD patients with deletions in the same region as the patients treated in our trial, higher levels of β-dystroglycan compared to α-sarcoglycan [4]

### Impact of treatment on levels of myofibre regeneration

We then demonstrated that 48-weeks treatment with golodirsen resulted in a 2.2% (− 5.9, 1.6 CI) average reduction in  % f/d myosin positive fibres. While the combined decreases in  % f/d myosin positive fibres between the 2 time points in the entire cohort were not statistically significant, natural history data suggests that without successful therapeutic intervention, over the same time period, an increase of +1.1% (− 1.1, 3.3 CI) f/d positive myofibres can be expected [44]. When considering these natural history findings, our data demonstrate a divergence of more than 3% between the 2 data points, a finding that suggests a biological effect of the AON on alleviating the cycles of degeneration and regeneration that occurs in DMD muscle.

In keeping with our hypothesis, we detected a significant negative correlation between levels of dystrophin (measured as both mean fluorescence intensity and  % dystrophin positive myofibres) and  % positive f/d myofibres. The majority of patients with high levels of dystrophin presented with the lowest level of regenerating fibres, further suggesting that there is a degree of molecular functionality of this induced protein. We indeed demonstrated a clear clustering of post-treatment biopsies with the highest level of dystrophin (by both measurements) and the lowest percentage of regenerating myofibres.

This is the first study in the human in which both increase in DAPC levels in dystrophin positive sarcolemma and reduction of  % f/d myosin positive fibres was demonstrated following a therapeutic intervention that induces production of dystrophin protein. It is important to emphasise that preclinical studies in both human and mouse have previously shown that very modest dystrophin levels, as little as 3%, can have a significant benefit to muscle function [27, 33, 36, 37, 42]. Additionally, it has been observed that the exon 44-amenable DMD population, who have residual low levels of trace dystrophin expression, experience a slower rate of disease progression than patients with other DMD mutations who lack these low protein levels [3, 5, 6, 38]. Previous studies have also demonstrated that despite BMD patients having marginally lower levels of f/d myosin positive fibres compared to DMD patients, they have significantly less severe phenotypes and improved clinical outcomes [14]. Therefore, our findings of a decrease in regeneration and changes in DAPC members following AON therapy is encouraging. Furthermore, our pathological study was after only 48-weeks therapeutic intervention. Previous work has shown that patients treated with a similar AON, eteplirsen, see a sustained accumulation of dystrophin over time with continual doses enabling eteplirsen to access more muscle fibres [7, 8, 29]. Preclinical studies in mice have also demonstrated a dose-related amelioration in pathology following an extended period of PMO therapeutic intervention [28]. It is therefore plausible for DMD patients treated chronically with golodirsen to experience a similar enhanced benefit from extended treatment, both in terms of dystrophin production and overall reduction in muscle pathology.

### Limitations

Whilst pre and post treatment sample pairs were blindly evaluated throughout the course of data acquisition and analysis, untreated sample pairs are not available for this study and thus and we are unable to compare with patients who did not receive therapeutic intervention.

Furthermore, whilst some evidence of the molecular functionality of induced dystrophin following a sustained period of therapeutic intervention is provided, it is important to note that dystrophin has multiple molecular functions, and only some were assessed in this study. Furthermore, the DAPC contains a multitude of proteins and protein binding interactions. Analysis of more DAPC members including β, γ and δ-sarcoglycan, α-dystroglycan and nNOS would be necessary to reveal more information about the comprehensive picture of DAPC restoration following treatment. As we have not studied markers of satellite cells, a theoretical possibility is that the treatment could have negatively influenced the regenerative capacity of the muscle in our patients. However, we notice that preclinical studies are concordant with our data and interpretation of findings, and that the significant correlation between dystrophin restoration and reduction in regenerative fibres argues against a generic toxic effect of the treatment. Finally, by utilising a cocktail of fetal and developmental myosin antibodies, we hoped to identify as many regenerating myofibres as possible. However, we cannot fully discriminate true regenerating fibres from those that have aberrantly reactivated these myosin isoforms. There is also a technical limitation of the image analysis to correctly identify and classify very small myofibres that preferentially express fetal myosin but not always developmental myosin.

### Conclusion

This is the first study to assess the effect on regeneration in human DMD muscle following a sustained period of successful therapeutic intervention with a dystrophin restoring therapy. We show that 48-weeks treatment with exon 53 skipping PMO golodirsen resulted in a significant negative correlation between the amount of dystrophin and levels of regeneration observed in different biopsy samples. We also documented positive correlations between the total and colocalised amounts of dystrophin and β-dystroglycan in post-treatment biopsies. Overall, these results support the indication of molecular functionality of the induced dystrophin following treatment with golodirsen. Future studies will be needed to correlate these findings to the clinical implication for patients of these improved pathological aspects as this will shed further light on the complex factors that impact the molecular functionality of induced dystrophin.

## Supplementary Information

**Additional file 1: Figure S1.**% a-sarcoglycan (a) and B-dystroglycan (b) positive myofibres at baseline and 48 weeks for each individual patient. Mean percentage α-sarcoglycan (c) and β-dystroglycan (d) positive myofibres at baseline and 48 weeks for all 25 patients. Fibres were considered positive if they demonstrated greater than 25% sarcolemmal circumference coverage for the protein in question. **Additional file 1: Figure S2.** Baseline and 48 weeks sarcolemmal fluorescence intensity for β-dystroglycan (a) and α-sarcoglycan (b) along with percentage change in fluorescence intensity for β-dystroglycan (c) and α-sarcoglycan (d) between the two time points. **Additional file 1: Figure S3.** (a) Average % change in f/d myosin positive fibres for patients that saw an increase, decrease or no change (+- 1%) between baseline and 48 week time points.Correlation between % f/d myosin positive after 48 weeks treatment with percentage change in dystrophin intensity (b) and percentage change in dystrophin positive myofibres (c) between baseline and 48 week time points.

## Data Availability

All data generated or analysed during this study are included in this published article.

## Abbreviations

AON: Antisense oligonucleotide
AU: Arbitrary units
BMD: Becker muscular dystrophy
ECM: Extracellular matrix
DAP: Dystrophin associated protein
DAPC: Dystrophin associated protein complex
DMD: Duchenne muscular dystrophy
F/D: Fetal and developmental
PMO: Phosphorodiamidate Morpholino Oligomer
WSI: Whole slide images

## References

[1] Aartsma-Rus A, Fokkema I, Verschuuren J, Ginjaar I, van Deutekom J, van Ommen G-J, den Dunnen JT (2009). Theoretic applicability of antisense-mediated exon skipping for Duchenne muscular dystrophy mutations. Hum Mutat.

[2] Aartsma-Rus A, Straub V, Hemmings R, Haas M, Schlosser-Weber G, Stoyanova-Beninska V, Mercuri E, Muntoni F, Sepodes B, Vroom E, Balabanov P (2017). Development of exon skipping therapies for duchenne muscular dystrophy: a critical review and a perspective on the outstanding issues. Nucleic Acid Ther.

[3] Anthony K, Arechavala-Gomeza V, Ricotti V, Torelli S, Feng L, Janghra N, Tasca G, Guglieri M, Barresi R, Armaroli A, Ferlini A, Bushby K, Straub V, Ricci E, Sewry C, Morgan J, Muntoni F (2014). Biochemical characterization of patients with in-frame or out-of-frame DMD deletions pertinent to exon 44 or 45 skipping. JAMA Neurol.

[4] Anthony K, Cirak S, Torelli S, Tasca G, Feng L, Arechavala-Gomeza V, Armaroli A, Guglieri M, Straathof CS, Verschuuren JJ, Aartsma-Rus A, Helderman-van den Enden P, Bushby K, Straub V, Sewry C, Ferlini A, Ricci E, Morgan JE, Muntoni F (2011). Dystrophin quantification and clinical correlations in Becker muscular dystrophy: implications for clinical trials. Brain.

[5] Bello L, Morgenroth LP, Gordish-Dressman H, Hoffman EP, McDonald CM, Cirak S (2016). DMD genotypes and loss of ambulation in the CINRG Duchenne natural history study. Neurology.

[6] Brogna C, Coratti G, Pane M, Ricotti V, Messina S, D’Amico A, Bruno C, Vita G, Berardinelli A, Mazzone E, Magri F, Ricci F, Mongini T, Battini R, Bello L, Pegoraro E, Baranello G, Previtali SC, Politano L, Comi GP, Sansone VA, Donati A, Bertini E, Muntoni F, Goemans N, Mercuri E, on behalf on the International DMD group (2019). Long-term natural history data in Duchenne muscular dystrophy ambulant patients with mutations amenable to skip exons 44, 45, 51 and 53. PLoS ONE.

[7] Charleston JS, Schnell FJ, Dworzak J, Donoghue C, Lewis S, Chen L, Young GD, Milici AJ, Voss J, DeAlwis U, Wentworth B, Rodino-Klapac LR, Sahenk Z, Frank D, Mendell JR (2018). Eteplirsen treatment for Duchenne muscular dystrophy: exon skipping and dystrophin production. Neurology.

[8] Cirak S, Arechavala-Gomeza V, Guglieri M, Feng L, Torelli S, Anthony K, Abbs S, Garralda ME, Bourke J, Wells DJ, Dickson G, Wood MJA, Wilton SD, Straub V, Kole R, Shrewsbury SB, Sewry C, Morgan JE, Bushby K, Muntoni F (2011). Exon skipping and dystrophin restoration in patients with Duchenne muscular dystrophy after systemic phosphorodiamidate morpholino oligomer treatment: an open-label, phase 2, dose-escalation study. Lancet.

[9] Cirak S, Feng L, Anthony K, Arechavala-Gomeza V, Torelli S, Sewry C, Morgan JE, Muntoni F (2012). Restoration of the dystrophin-associated glycoprotein complex after exon skipping therapy in duchenne muscular dystrophy. Mol Ther.

[10] Claflin DR, Brooks SV (2008). Direct observation of failing fibers in muscles of dystrophic mice provides mechanistic insight into muscular dystrophy. Am J Physiol Cell Physiol.

[11] Clemens PR, Rao VK, Connolly AM, Harper AD, Mah JK, Smith EC, McDonald CM, Zaidman CM, Morgenroth LP, Osaki H, Satou Y, Yamashita T, Hoffman EP, Investigators CINRGDNHS (2020). Safety, tolerability, and efficacy of viltolarsen in boys with duchenne muscular dystrophy amenable to exon 53 skipping: a phase 2 randomized clinical trial. JAMA Neurol.

[12] Constantin B (2014). Dystrophin complex functions as a scaffold for signalling proteins. Biochimica et Biophysica Acta (BBA) Biomembranes.

[13] Duan D (2018). Systemic AAV micro-dystrophin gene therapy for duchenne muscular dystrophy. Mol Ther.

[14] Dubowitz V, Oldfors A, Sewry CA (2020) Muscle biopsy: a practical approach

[15] Ervasti JM (2003). Costameres: the Achilles’ Heel of Herculean muscle. J Biol Chem.

[16] Ervasti JM (2013). Structure and function of the dystrophin-glycoprotein complex.

[17] Finkel RS, Flanigan KM, Wong B, Bönnemann C, Sampson J, Sweeney HL, Reha A, Northcutt VJ, Elfring G, Barth J, Peltz SW (2013). Phase 2a study of ataluren-mediated dystrophin production in patients with nonsense mutation Duchenne muscular dystrophy. PLoS ONE.

[18] Flanigan KM (2014). Duchenne and Becker muscular dystrophies. Neurol Clin.

[19] Frank DE, Schnell FJ, Akana C, El-Husayni SH, Desjardins CA, Morgan J, Charleston JS, Sardone V, Domingos J, Dickson G, Straub V, Guglieri M, Mercuri E, Servais L, Muntoni F (2020). Increased dystrophin production with golodirsen in patients with Duchenne muscular dystrophy. Neurology.

[20] Gao QQ, McNally EM (2015). The dystrophin complex: structure, function, and implications for therapy. Compr Physiol.

[21] Guiraud S, Davies KE (2019). Regenerative biomarkers for Duchenne muscular dystrophy. Neural Regener Res.

[22] Guiraud S, Edwards B, Squire SE, Moir L, Berg A, Babbs A, Ramadan N, Wood MJ, Davies KE (2019). Embryonic myosin is a regeneration marker to monitor utrophin-based therapies for DMD. Hum Mol Genet.

[23] Gumerson JD, Michele DE (2011). The dystrophin-glycoprotein complex in the prevention of muscle damage. J Biomed Biotechnol.

[24] Hoffman EP, Brown RH, Kunkel LM (1987). Dystrophin: the protein product of the Duchenne muscular dystrophy locus. Cell.

[25] Janghra N, Morgan JE, Sewry CA, Wilson FX, Davies KE, Muntoni F, Tinsley J (2016). Correlation of utrophin levels with the dystrophin protein complex and muscle fibre regeneration in duchenne and becker muscular dystrophy muscle biopsies. PLoS ONE.

[26] Koenig M, Beggs AH, Moyer M, Scherpf S, Heindrich K, Bettecken T, Meng G, Müller CR, Lindlöf M, Kaariainen H, de la Chapelle A, Kiuru A, Savontaus M-L, Gilgenkrantz H, Récan D, Chelly J, Kaplan J-C, Covone AE, Archidiacono N, Romeo G, Liechti-Gallati S, Schneider V, Braga S, Moser H, Darras BT, Murphy P, Francke U, Chen JD, Morgan G, Denton M, Greenberg CR, Wrogemann K, Blonden LAJ, van Paassen HMB, van Ommen GJB, Kunkel LM (1989). The molecular basis for Duchenne versus Becker muscular dystrophy: correlation of severity with type of deletion. Am J Hum Genet.

[27] Li D, Yue Y, Duan D (2008). Preservation of muscle force in Mdx3cv mice correlates with low-level expression of a near full-length dystrophin protein. Am J Pathol.

[28] Malerba A, Sharp PS, Graham IR, Arechavala-Gomeza V, Foster K, Muntoni F, Wells DJ, Dickson G (2011). Chronic systemic therapy with low-dose morpholino oligomers ameliorates the pathology and normalizes locomotor behavior in mdx mice. Mol Ther.

[29] Mendell JR, Rodino-Klapac LR, Sahenk Z, Roush K, Bird L, Lowes LP, Alfano L, Gomez AM, Lewis S, Kota J, Malik V, Shontz K, Walker CM, Flanigan KM, Corridore M, Kean JR, Allen HD, Shilling C, Melia KR, Sazani P, Saoud JB, Kaye EM, Eteplirsen Study Group (2013). Eteplirsen for the treatment of Duchenne muscular dystrophy. Ann Neurol.

[30] Mendell JR, Shilling C, Leslie ND, Flanigan KM, Al-Dahhak R, Gastier-Foster J, Kneile K, Dunn DM, Duval B, Aoyagi A, Hamil C, Mahmoud M, Roush K, Bird L, Rankin C, Lilly H, Street N, Chandrasekar R, Weiss RB (2012). Evidence-based path to newborn screening for Duchenne muscular dystrophy. Ann Neurol.

[31] Meng J, Counsell JR, Reza M, Laval SH, Danos O, Thrasher A, Lochmüller H, Muntoni F, Morgan JE (2016). Autologous skeletal muscle derived cells expressing a novel functional dystrophin provide a potential therapy for Duchenne muscular dystrophy. Sci Rep.

[32] Meng J, Muntoni F, Morgan J (2018). CD133 + cells derived from skeletal muscles of Duchenne muscular dystrophy patients have a compromised myogenic and muscle regenerative capability. Stem Cell Res.

[33] Neri M, Torelli S, Brown S, Ugo I, Sabatelli P, Merlini L, Spitali P, Rimessi P, Gualandi F, Sewry C, Ferlini A, Muntoni F (2007). Dystrophin levels as low as 30% are sufficient to avoid muscular dystrophy in the human. Neuromuscul Disord.

[34] Omairi S, Hau K-L, Collin-Hooper H, Montanaro F, Goyenvalle A, Garcia L, Patel K (2017). Link between MHC fiber type and restoration of dystrophin expression and key components of the DAPC by tricyclo-DNA-mediated exon skipping. Mol Ther Nucleic Acids.

[35] Potaczek DP, Garn H, Unger SD, Renz H (2016). Antisense molecules: a new class of drugs. J Allergy Clin Immunol.

[36] van Putten M, Hulsker M, Nadarajah VD, van Heiningen SH, van Huizen E, van Iterson M, Admiraal P, Messemaker T, den Dunnen JT, ’t Hoen PAC, Aartsma-Rus A (2012). The effects of low levels of dystrophin on mouse muscle function and pathology. PLoS ONE.

[37] van Putten M, Hulsker M, Young C, Nadarajah VD, Heemskerk H, van der Weerd L, ’t Hoen PAC, van Ommen GJB, Aartsma-Rus AM (2013). Low dystrophin levels increase survival and improve muscle pathology and function in dystrophin/utrophin double-knockout mice. FASEB J.

[38] Ricotti V, Ridout DA, Pane M, Main M, Mayhew A, Mercuri E, Manzur AY, Muntoni F, UK NorthStar Clinical Network (2016). The NorthStar ambulatory assessment in Duchenne muscular dystrophy: considerations for the design of clinical trials. J Neurol Neurosurg Psychiatry.

[39] Sardone V, Zhou H, Muntoni F, Ferlini A, Falzarano MS (2017). Antisense oligonucleotide-based therapy for neuromuscular disease. Molecules.

[40] Scaglioni D, Ellis M, Catapano F, Torelli S, Chambers D, Feng L, Sewry C, Morgan J, Muntoni F, Phadke R (2020). A high–throughput digital script for multiplexed immunofluorescent analysis and quantification of sarcolemmal and sarcomeric proteins in muscular dystrophies. Acta Neuropathol Commun.

[41] Schiaffino S, Rossi AC, Smerdu V, Leinwand LA, Reggiani C (2015). Developmental myosins: expression patterns and functional significance. Skelet Muscle.

[42] Sharp PS, Bye-a-Jee H, Wells DJ (2011). Physiological characterization of muscle strength with variable levels of dystrophin restoration in mdx mice following local antisense therapy. Mol Ther.

[43] Shimizu-Motohashi Y, Murakami T, Kimura E, Komaki H, Watanabe N (2018). Exon skipping for Duchenne muscular dystrophy: a systematic review and meta-analysis. Orphanet J Rare Dis.

[44] Tinsley J, Muntoni F, Layton G, Faelan C, Patterson-Kane J, Hetherington A, Davies K (2018). DMD clinical therapies II: P.132 identification of developmental myosin positive fibres acts both as a clinical biomarker for muscle disease and an important component of the process to confirm ezutromid target engagement. Neuromuscul Disord.

[45] Wang B, Li J, Xiao X (2000). Adeno-associated virus vector carrying human minidystrophin genes effectively ameliorates muscular dystrophy in mdx mouse model. PNAS.

[46] Watanabe N, Nagata T, Satou Y, Masuda S, Saito T, Kitagawa H, Komaki H, Takagaki K, Takeda S (2018). NS-065/NCNP-01: an antisense oligonucleotide for potential treatment of exon 53 skipping in Duchenne muscular dystrophy. Mol Ther Nucleic Acids.

[47] van Westering TLE, Lomonosova Y, Coenen-Stass AML, Betts CA, Bhomra A, Hulsker M, Clark LE, McClorey G, Aartsma-Rus A, van Putten M, Wood MJA, Roberts TC (2020). Uniform sarcolemmal dystrophin expression is required to prevent extracellular microRNA release and improve dystrophic pathology. J Cachexia Sarcopenia Muscle.

[48] Yiu EM, Kornberg AJ (2015). Duchenne muscular dystrophy. J Paediatr Child Health.

[49] Yoshimura M, Sakamoto M, Ikemoto M, Mochizuki Y, Yuasa K, Miyagoe-Suzuki Y, Takeda S (2004). AAV vector-mediated microdystrophin expression in a relatively small percentage of mdx myofibers improved the mdx phenotype. Mol Ther.

