# Supplementary material for: The administration of antisense oligonucleotide golodirsen reduces pathological regeneration in patients with Duchenne muscular dystrophy
Source: Acta Neuropathol Commun. 2021 Jan 6;9:7. doi: 10.1186/s40478-020-01106-1 (PMC7789286; doi:10.1186/s40478-020-01106-1)
Supplement: Supplementary file 1 — Additional file 1: Figure S1.% a-sarcoglycan (a) and B-dystroglycan (b) positive myofibres at baseline and 48 weeks for each individual patient. Mean percentage α-sarcoglycan (c) and β-dystroglycan (d) positive myofibres at baseline and 48 weeks for all 25 patients. Fibres were considered positive if they demonstrated greater than 25% sarcolemmal circumference coverage for the protein in question. Additional file 1: Figure S2. Baseline and 48 weeks sarcolemmal fluorescence intensity for β-dystroglycan (a) and α-sarcoglycan (b) along with percentage change in fluorescence intensity for β-dystroglycan (c) and α-sarcoglycan (d) between the two time points. Additional file 1: Figure S3. (a) Average % change in f/d myosin positive fibres for patients that saw an increase, decrease or no change (+- 1%) between baseline and 48 week time points.Correlation between % f/d myosin positive after 48 weeks treatment with percentage change in dystrophin intensity (b) and percentage change in dystrophin positive myofibres (c) between baseline and 48 week time points. [file 40478_2020_1106_MOESM1_ESM.docx]

**Supplementary Figures**

**
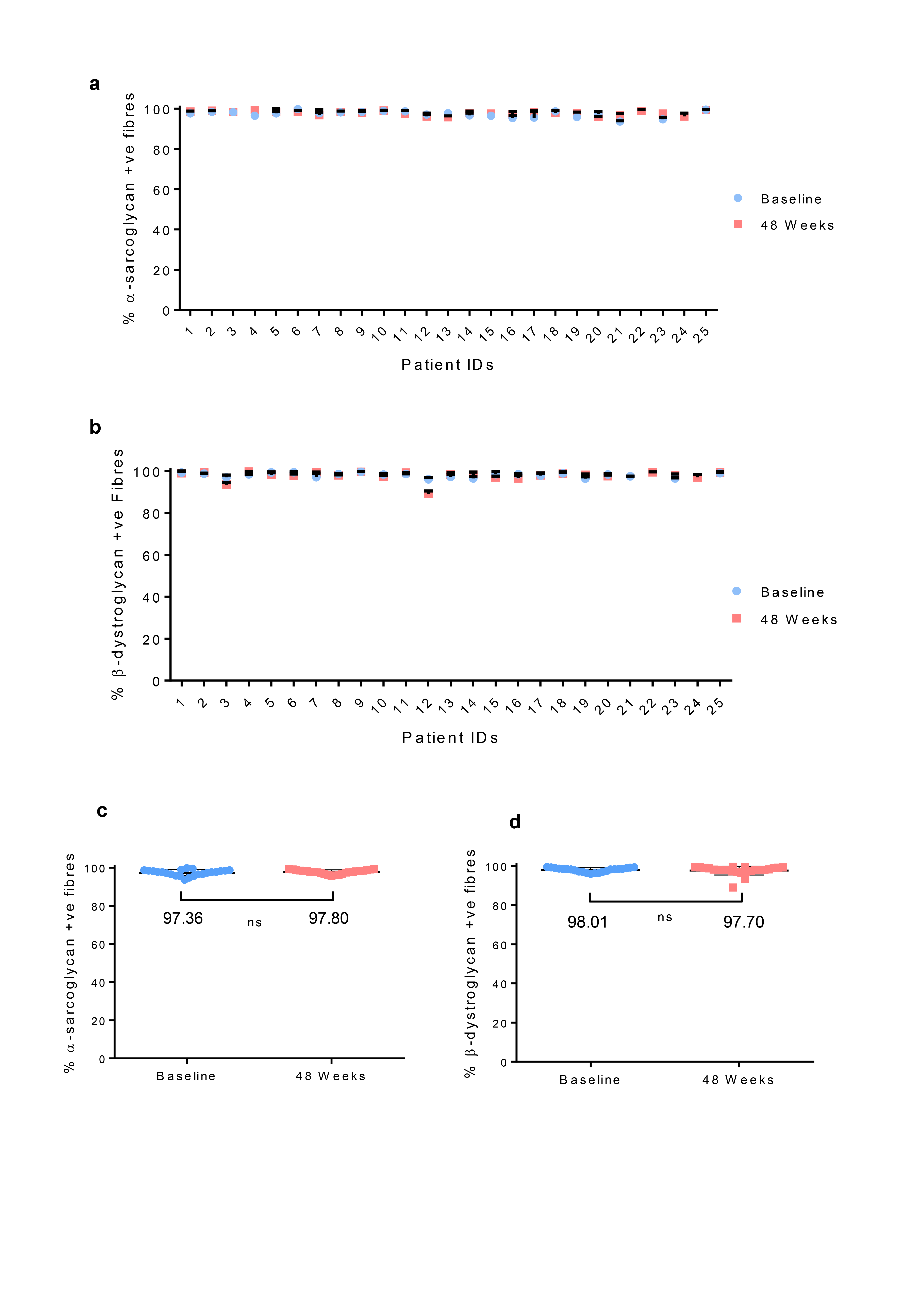
**

**Fig.S1**

*% a-sarcoglycan* ***(a)*** *and B-dystroglycan* ***(b)*** *positive myofibres at baseline and 48 weeks for each individual patient. Mean percentage α-sarcoglycan* ***(c)*** *and β-dystroglycan* ***(d)*** *positive myofibres at baseline and 48 weeks for all 25 patients. Fibres were considered positive if they demonstrated greater than 25% sarcolemmal circumference coverage for the protein in question.*

**
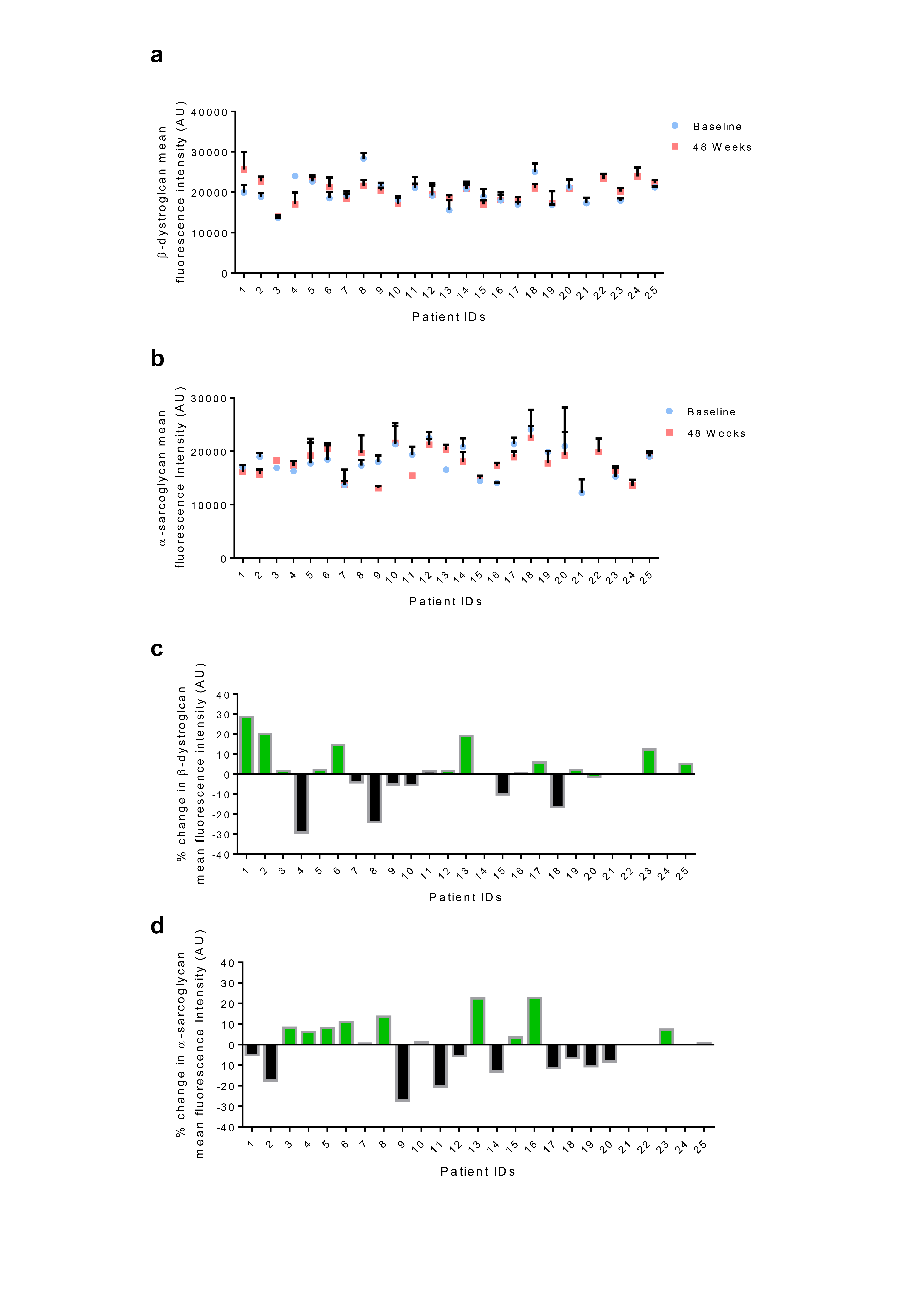
**

**Fig.S2**

*Baseline and 48 weeks sarcolemmal fluorescence intensity for β-dystroglycan* ***(a)*** *and α-sarcoglycan* ***(b)*** *along with percentage change in fluorescence intensity for β-dystroglycan* ***(c)*** *and α-sarcoglycan* ***(d)*** *between the two time points*

**
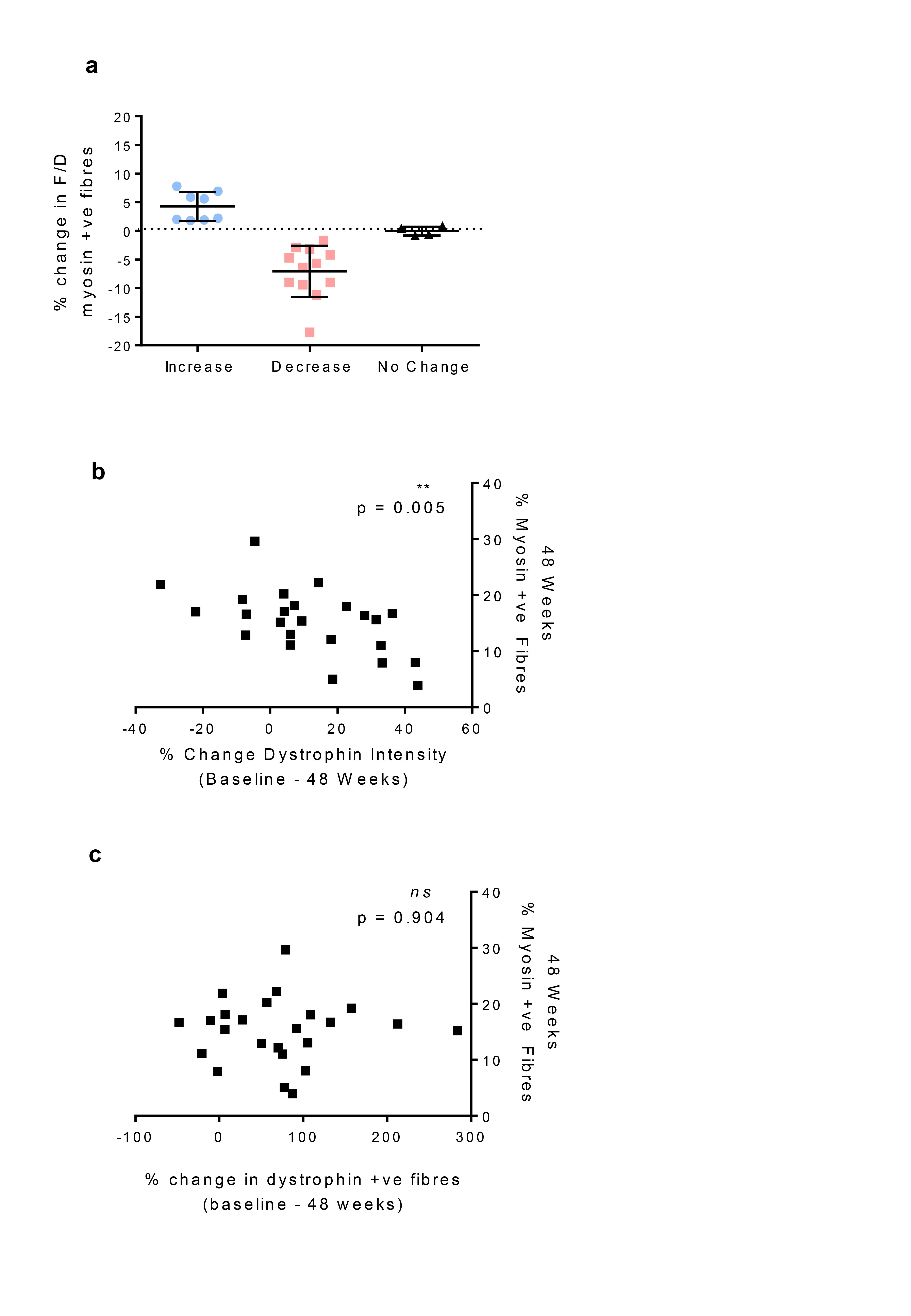
**

**Fig.S3**

***(a)*** *Average % change in f/d myosin positive fibres for patients that saw an increase, decrease or no change (+- 1%) between baseline and 48 week time points.*

*Correlation between % f/d myosin positive after 48 weeks treatment with percentage change in dystrophin intensity* ***(b)*** *and percentage change in dystrophin positive myofibres* ***(c)*** *between baseline and 48 week time points.*
